# Non-invasive screening for liver fibrosis by acoustic radiation force impulse in patients with ciliopathies

**DOI:** 10.1038/s41598-025-96246-6

**Published:** 2025-04-17

**Authors:** Johanna Bresch, Jens König, Martin Konrad, Sabine Kollmann, Mareike Dahmer-Heath, Hauke Sebastian Heinzow, Michael Praktiknjo, Jonel Trebicka, Carsten Bergmann, Hartmut H-J Schmidt, Bernhard Schlevogt

**Affiliations:** 1 Department of Medicine B, Muenster University Hospital, Albert-Schweitzer-Campus 1, Building A14, 48149 Muenster, Germany; 2 Department of General Pediatrics, Muenster University Hospital, Muenster, Germany; 3 Brüderkrankenhaus Trier, Trier, Germany; 4 Medizinische Genetik Mainz, Limbach Genetics, Mainz, Germany; 5 Department of Medicine IV, Faculty of Medicine, Medical Center-University of Freiburg, Freiburg, Germany; 6 Department of Gastroenterology, Hepatology and Transplant Medicine, University Hospital Essen, Essen, Germany; 7 Department of Gastroenterology and Endoscopy, Medical Center Osnabrueck, Osnabrueck, Germany

**Keywords:** Ciliopathies, Congenital hepatic fibrosis, Liver elastography, Liver stiffness measurement, Spleen stiffness measurement, Liver fibrosis, Ultrasonography, Paediatric kidney disease, Polycystic kidney disease

## Abstract

Primary cilia are antenna-like structures on the surface of epithelial cells involved in multiple signaling pathways. Their malfunction can cause a heterogenous group of diseases called ciliopathies with a broad spectrum of organ involvements, including liver fibrosis. The aim of this exploratory study was to evaluate elastography measurement via ultrasound based acoustic radiation force impulse imaging (ARFI) as a screening tool for liver fibrosis in ciliopathies. In a prospective cohort of 51 patients with ciliopathies (aged between 2 months and 66 years) from the NEOCYST registry stiffness of the right liver lobe and spleen was measured via ARFI and results were then compared with laboratory parameters, endoscopic, ultrasonographic and histological findings. ARFI screening of the liver identified 27 patients without increased liver stiffness suggesting no or insignificant fibrosis, 11 with intermediate fibrosis, and 12 with liver fibrosis F4. Four patients showed increased spleen stiffness in ARFI. In all 10 patients with histologically confirmed fibrosis, ARFI results perfectly matched fibrosis stages. In the ARFI-based overall fibrosis subgroup, ALT, AST, GGT and spleen size were significantly increased, whereas platelets were significantly decreased compared to the no fibrosis subgroup. Normal GGT excluded ARFI-defined F4 fibrosis (negative predictive value 100%). Gene variants in *PKHD1*, *TMEM67*, and *TULP3* were primarily detected in our patients with liver fibrosis whereas *NPHP1* and *HNF1B* were not associated with increased liver stiffness. ARFI is a valuable screening tool for the detection of liver involvement in ciliopathies and may be useful in addition to laboratory and clinical parameters alone.

Trial registration: NEOCYST registry DRKS00011003, registered 06.09.2016, https://drks.de/search/en/trial/DRKS00011003.

## Background

Ciliopathies are rare genetic disorders comprising a wide variety of syndromes and symptom complexes, caused by hereditary malfunction of cilia. Generally, primary immotile cilia and motile cilia are distinguished by their structure^1^. Motile cilia mostly mediate fluid transportation in the lower respiratory tract, the central nervous system and other liquid containing compartments whereas immotile cilia play an invaluable role in the conversion of extracellular stimuli (e.g. mechanosensation, chemosensation) into a cellular response (e.g. proliferation, differentiation) and thus are essential for normal tissue function^2^. From a genetic point of view, variants in genes that encode proteins affecting either the structure or functionality of motile and immotile (primary) cilia are known to cause ciliopathic disorders, implying their locus heterogeneity^3^.

As primary cilia are ubiquitously present, many different organ systems can be impaired by their dysfunction, comprising cystic kidney malformations, tapetoretinal disorders, brain malformations and ductal plate malformations resulting in congenital hepatic fibrosis^2^. Due to the ultrarare occurrence of the individual disease entities and gene variants as well as extensive locus heterogeneity, accumulation of large cohorts with well-defined genotypes and phenotypes is problematic.

Ductal plate malformation is a defective remodeling of ductal plate during bile duct morphogensis resulting in congenital hepatic fibrosis^4^. This is the proposed mechanism of hepatic injury in most ciliopathies such as autosomal recessive polycystic kidney disease (ARPKD), Meckel-Gruber syndrome (MGS) or some forms of Joubert syndrome. However, it can also be observed in patients with certain subtypes of nephronophthisis or Bardet-Biedl syndrome (BBS)^5^. Only recently, *TULP*3-associated ciliopathy was described with an obligate liver involvement^6^. Ciliopathy-associated liver disease can lead to serious complications such as portal hypertension (PH) with an increased risk for variceal bleeding^5^. Thus, early detection of liver fibrosis is crucial for patients with increased risk for development of fibrosis. Due to genetic and allelic heterogeneity, the small number of patients and variable disease expression robust genotype-phenotype correlations implying risk prognostication are still challenging. Consequentially, additional screening tools and biomarkers detecting hepatic fibrosis as well as clinically significant portal hypertension are needed.

Initial liver fibrosis screening is often done by conventional ultrasound and evaluation of serum markers such as APRI (AST-Platelet Ratio Index) and FIB-4 (AST, ALT, age, platelet count). However, both methods only inadequately identify less advanced fibrosis stages as ultrasound fibrosis signs may be missing in intermediate fibrosis stages or early cirrhosis^7,8^. Both APRI and FIB-4 are influenced by a low platelet count which is a surrogate marker of portal hypertension, reducing its ability to detect early fibrosis stages in which portal hypertension may be absent.

Acoustic radiation force impulse imaging (ARFI) is a well-established, non-invasive method for the determination of tissue elasticity integrated in many modern ultrasound machines. Briefly, acoustic pulses are exciting a local tissue displacement which results in the measurable propagation of waves transversal to the initial pulse. The velocity of these waves can be measured by ultrasound waves and is a function of tissue stiffness^9^. Increased ARFI velocities reliably correlate with liver fibrosis^10,11^. Furthermore, splenic ARFI has been shown to identify patients with esophageal varices^12,13^. Considering that traditional screening methods such as sonography or serum markers only inadequately identify fibrotic changes of the liver^14^the aim of this study was to evaluate ARFI as a non-invasive screening tool in patients with ciliopathies. Since several studies have already shown the suitability of ARFI as a surrogate marker of liver fibrosis in various pediatric patient populations^15–18^ this approach appeared promising for patients suffering from ciliopathies.

## Methods

For this prospective study, patients treated at Muenster University Hospital were recruited from the NEOCYST (Network for early onset cystic kidney disease) registry. The NEOCYST registry is a clinical and genetic database prospectively acquiring data from care units specialized in ciliopathies to improve the understanding and management of rare ciliopathies (www.neocyst.de)^19^. Informed written consent was obtained from all participants or parents/legal guardians and data was obtained with consent of the institutional ethics commission (AZ2016-284-f-S).

### Participants

Those eligible were individuals with suspected renal ciliopathies (nephronophthisis-related phenotype (NPH-RC), Joubert syndrome spectrum (JS), autosomal recessive polycystic kidney disease (ARPKD)) diagnosed by either the clinical presentation or the presence of disease-causing variants in ciliary genes. The clinical diagnosis of NPH was based on at least two of the following criteria: (1) characteristic clinical course with polyuria/polydipsia, (2) chronic kidney disease (CKD), (3) kidney ultrasound or biopsy suggestive of NPH-RC, and (4) pedigree compatible with autosomal recessive inheritance. Neurological criteria for Joubert syndrome were based on the presence of a molar tooth sign on magnetic resonance imaging or the clinical diagnosis. COACH syndrome was diagnosed if additional hepatic fibrosis and/or ocular coloboma were found.

For genetic confirmation, PCR-based gel electrophoresis was applied for the detection of a homozygous *NPHP1* deletion. In others, targeted Sanger sequencing, a specific ciliopathy multigene panel analysis described in detail elsewhere^6^ or a whole exome sequencing approach were used based on the patient’s phenotype. Variants were classified according to diagnostic criteria of the American College of Medical genetics and Genomics (ACMG)^20^. Besides class V and IV variants, variants of unknown significance were only reported if they had a high probability to be causative (hot/warm/tepid)^21^.

### Interpretation of clinical data

Apart from histologically confirmed fibrosis (according to Batts-Ludwig fibrosis staging), potential clinical signs for liver involvement were considered elevated alanine aminotransferase (ALT) and/or aspartate transaminase (AST), hepatomegaly or portal hypertension, the latter being indicated by splenomegaly, thrombocytopenia, or esophageal varices in esophagogastroduodenoscopy (EGD). ALT and AST were defined elevated when exceeding 35 U/l for adult females and 50 U/l for adult males. Gamma-glutamyl transferase (GGT) elevation for adult males and females was defined > 66 U/l and > 39 U/l. Pediatric age-adjusted reference values of the local laboratory were between 30 and 65 U/l for ALT, between 30 and 100 U/l for AST and between 5 and 39 U/l for GGT, respectively. Thrombocytopenia was defined as platelet counts below 150 × 10^9^/l. Splenomegaly and hepatomegaly were defined as organ lengths above 97.5th percentiles for height^22^.

### Conduction of elastography

The examination was conducted using the convex transducer (1–4 MHz) of an Acuson S2000 ultrasound scanner (Siemens Healthcare GmbH, Erlangen, Germany) and based on the EFSUMB guidelines^23^. In a calm environment, the fasting patients were asked to elevate their arms while resting in supine position.

Under intercostal sight in B-mode, the “region of interest” was placed in an area of organ tissue free of vessels or bile ducts 1–2 cm below the organ capsules. Ten measurements each in liver segments 6 and 7 and spleen were performed in virtual touch mode. The measurements were taken during breath-hold at a mean respiratory level in cooperating patients, for others in respiratory rest position. Median and interquartile range (IQR) of each measuring site were calculated. Median segment measurements with IQR / median ratio > 30% were excluded and IQR / median ratio < 30% in at least one liver segment served as inclusion criterium. Patients with 2 invalid segment measurements were excluded.

### ARFI reference values

ARFI measurements of the participants were compared to reference values of healthy pediatric and adult individuals^24,25^. If valid measurements were obtained in both liver segments, their mean was calculated. By their ARFI velocity cut-off values, the results were grouped into three categories: Normal liver stiffness (no fibrosis / insignificant fibrosis) was defined as ARFI velocity below 1.34 m/s. As there are no established cut-off values for F1 fibrosis in adults, this category summarized F0 in children and both F0 – F1 in adults. Consequently, intermediate fibrosis included F1 – F3 in children (≥ 1.34 m/s and < 2.13 m/s) and F2 – F3 in adults (≥ 1.34 m/s and < 1.8 m/s). Liver stiffness values exceeding intermediate fibrosis were classified F4 fibrosis (F4 ≥ 1.8 m/s in adults and ≥ 2.13 m/s in children). Cut-off values for spleen stiffness determined by *Takuma et al.*served as references for the presence of esophageal varices in adults, indicating PH for ARFI values > 3.18 m/s (high risk varices > 3.30 m/s)^26^. As pediatric cut-off values for the presence of esophageal varices were not available, the values referred to above were applied to children as well. Patients with F4 fibrosis were offered further care at the hepatology section.

ARFI measurement results were then compared with clinical characteristics, including laboratory parameters, sonographic, endoscopic and histological findings. For significance tests, patients with intermediate and F4 fibrosis were subsumed under “overall fibrosis” (F1/F2 - F4) and tested against patients with no fibrosis (F0/F1).

Statistical significances of cohort characteristics were calculated using two-sided t-tests for metric variables and X^2^-test for nominal variables. For comparison of laboratory parameters with ARFI readings contingency tables and measurements of diagnostic accuracy were calculated. Significances were tested by Fisher’s exact test. Diagnostic accuracy was further visualized by ROC curves and AUROC values. P-values < 0.05 were considered significant. Statistics were performed using Microsoft Excel 2019 ^®^ 365 32-Bit (Microsoft Corporation, Redmont, WA, USA). ROC and AU-ROC were calculated using XLSTAT 2023.2 (Lumivero, Denver, CO, USA).

## Results

### Study cohort

While one patient had to be excluded due to invalid ARFI measurements, inclusion criteria were met by 50 patients treated at Muenster University Hospital with an age range of 2 months to 66 years (mean 20.3 years) and 32% females. About half of the patients (48%) were children (Table 1). Diagnostic genetic variants were identified in 88% of patients (Table 1). Five groups of siblings shared familiar variants (*BBS7*, *NPHP5*, *PKHD1* and two groups of siblings with *TULP3* variants). 6 patients (12%) remained genetically unsolved. Two of these patients displayed a Joubert syndrome phenotype, the four others were characterized by the presence of corticomedullary kidney cysts and progressive kidney failure. All six genetically unsolved patients proceeded to end-stage kidney disease and received kidney transplantation. Overall, half of the participants required renal replacement therapy (dialysis and/or kidney transplantation) and 12% had clinically overt portal hypertension (esophageal varices or portosystemic shunt procedure).

**Table 1:** Clinical, genetic and laboratory characteristics of 50 patients with ciliopathies.

Cohort characteristics stratified by fibrosis stage based on ARFI elastography		All patients	No fibrosis/insignificant fibrosis	Intermediate fibrosis	F4 fibrosis	Overall fibrosis	p-value
			Adults F0 - F1	Adults F2 - F3	F4	Adults F2 - F4	t-test/ χ^2^ test
			Children F0	Children F1 - F3		Children F1 - F4	
		100% (n = 50)	54% (n = 27)	22% (n = 11)	24% (n = 12)	46% (n = 23)	
Age	Age [years, mean ± SD]	20.3 (15.7)	14.6 (11.1)	17.1 (9.9)	36.2 (18.1)	28 (19)	0.005
	Children [%]	48	34	12	2	14	0.023
	Children [years, mean ± SD]	8.2 (5.2)	7.7 (5.8)	9.2 (2.8)	10.5 (0)	9.4 (2.6)	0.478
	Adults [years, mean ± SD]	31.5 (13.9)	26.3 (7.6)	26.5 (6.8)	38.5 (17.1)	34.8 (15.7)	0.138
Sex	Female [%]	32	18	6	8	14	0.73
Laboratory parameters	AST [U/l, mean ± SD]	52 (38)	37 (27)	64 (43)	75 (38)	70 (41)	0.001
	ALT [U/l, mean ± SD]	59 (65)	38 (50)	87 (86)	79 (55)	83 (72)	0.015
	GGT [U/l, mean ± SD]	105 (127)	47 (98)	146 (135)	197 (109)	172 (125)	< 0.001
	AP [U/l, mean ± SD]	231 (167)	205 (165)	249 (159)	271 (268)	261 (164)	0.251
	APRI score [U x nl/l, mean ± SD]	0.84 (1.25)	0.32 (0.26)	0.59 (0.37)	2.25 (1.88)	1.45 (1.61)	< 0.001
	Platelets [1000/ml, mean ± SD]	230 (108)	268 (75)	266 (117)	115 (73)	187 (122)	0.007
Clinical data	Renal replacement therapy [%]	50	26	10	14	24	0.371
	Hepatomegaly [%], n = 47	36	11	9	19	28	0.016
	Splenomegaly [%], n = 47	54	19	13	26	38	0.003
	Clinical overt portal hypertension [%]	12	0	0	12	12	< 0.001
Genetic variants	Genetically diagnosed [%]	88	48	16	24	40	_
	HNF1B [%]	14	14	0	0	0	_
	NPHP1 [%]	12	12	0	0	0	_
	PKHD1 [%]	12	4	2	6	8	_
	TMEM67 [%]	16	2	6	8	14	_
	TULP3 [%]	12	0	2	10	12	_
	Other genes [%]	22	16	6	0	6	_
	Clinically diagnosed [%]	12	6	6	0	6	_

Parameters are stratified by ARFI liver fibrosis stage. For some clinical findings, data could not be obtained for all patients, therefore the percentage was calculated using the total number of patients with data available for these categories. ARFI values exceeding 1.34 m/s in liver were assessed as indicators for liver fibrosis (equaling F2 - F4 fibrosis in adults and F1 - F4 fibrosis in children). Statistical significances between *no fibrosis* and overall *fibrosis* subgroup for percentages of children, sex and clinical data were calculated using χ^2^ tests. For all other parameters, t-tests were performed. SD: standard deviation.

### Fibrosis screening with liver ARFI

**Fig. 1:**
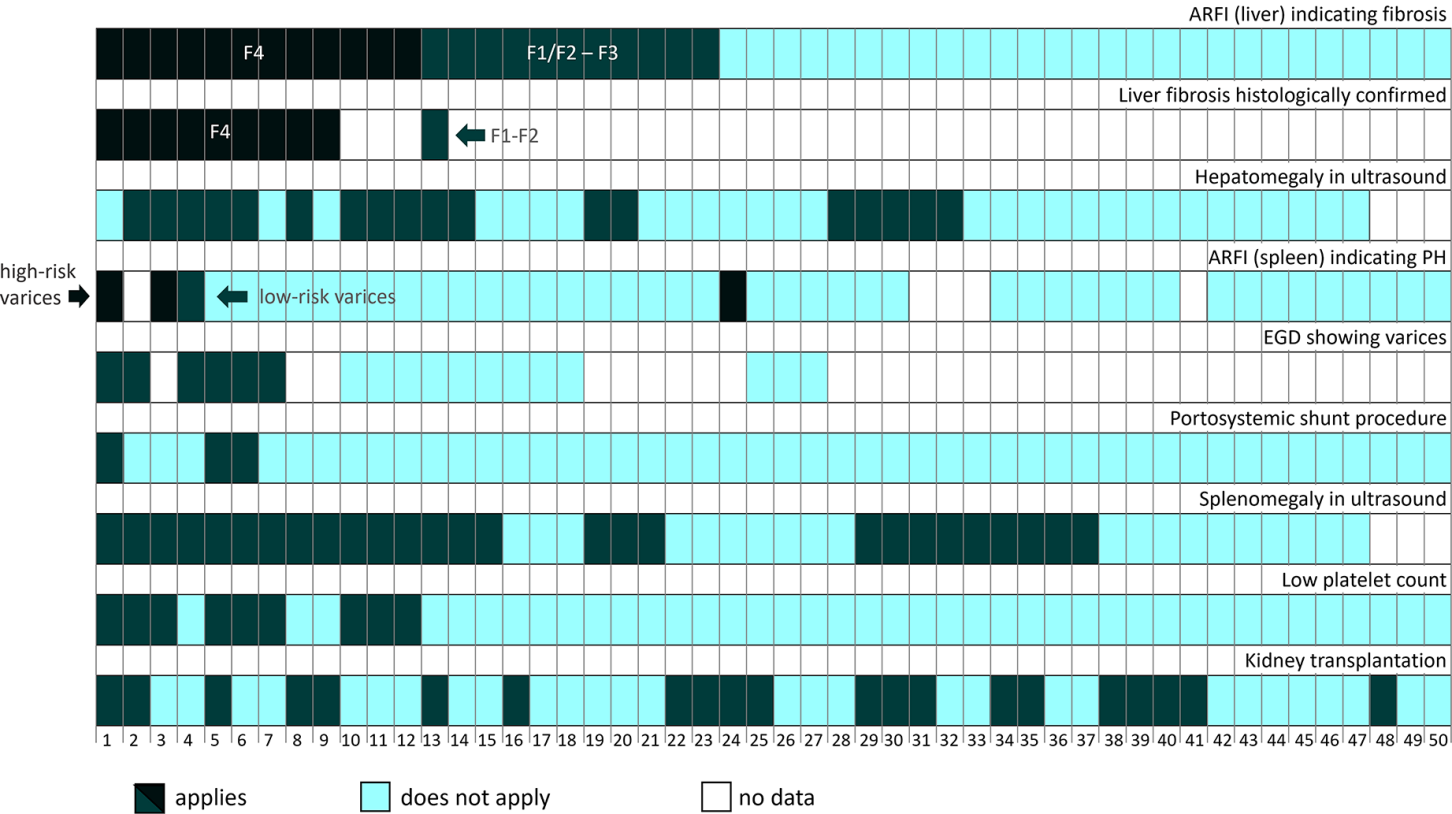
Individual association of ARFI velocities of liver and spleen with histological, clinical and imaging parameters for liver fibrosis and portal hypertension in patients with ciliopathies. Each column indicates one patient’s data. Portosystemic shunt procedure includes transjugular intrahepatic portosystemic shunt and shunt surgery.

For 84% of patients, 10 valid ARFI values were obtained in two different liver segments and therefore the mean of their medians was calculated. For the remaining 16%, one valid ARFI segmental measurement was available. One patient had to be excluded from the study due to two invalid segment measurements. ARFI imaging of the liver revealed 24% of patients with F4 fibrosis, 22% with intermediate fibrosis (adults F2 - F3, children F1 - F3) and 54% with normal liver stiffness (adults F0 – F1, children F0). Accordingly, 46% of patients were classified as “overall fibrosis” containing all patients with F4 fibrosis and intermediate fibrosis (Table 1). Liver histology results - the gold standard for the detection of liver fibrosis - were available for 9/12 patients with F4 fibrosis in ARFI and F4 fibrosis (Batts-Ludwig) was histologically confirmed for all of these participants (Pat. 1–9, Fig. 1). In patient 13 with F2 fibrosis in ARFI, previous liver biopsy had shown F1 - F2 fibrosis (Fig. 1). Thus, all patients with histologically confirmed fibrosis were identified by liver ARFI. 4 patients had their liver biopsy after ARFI measurement (mean 7.9 months, SD 11.1 months). 6 patients had their liver biopsy before ARFI measurement (mean 88.1 months, SD 74.2 months). Liver biopsy revealed F4 fibrosis in 5 of them which was later confirmed by ARFI. In patient 13, F1/F2 fibrosis was detected by biopsy and also confirmed by ARFI 7 years later.

### Detection of esophageal varices by Splenic ARFI

11 patients had their EGD before ARFI measurement (mean 4.8 months, SD 14.7 months). 8 patients had their EGD after ARFI measurement (mean 5.0 months, SD 3.7 months). Four patients (8%) with increased spleen stiffness were identified, three with F4 fibrosis in liver ARFI (Fig. 1). Those three patients showed splenomegaly (Pat. 1,3 and 4) and two of them had esophageal varices in EGD (Pat. 1 and 4) as clinical indicators of portal hypertension (Fig. 1). One patient was classified “high risk for variceal bleeding” in the ARFI examination of the spleen despite no evidence for portal hypertension on regular ultrasound and unremarkable ARFI results of the liver (Pat. 24). In three patients, spleen stiffness measurement was performed after treatment of portal hypertension with either a transjugular intrahepatic portosystemic shunt (TIPS) or a Warren shunt (Pat. 1, 5 and 6). In two of them ARFI results revealed spleen stiffness levels within normal ranges, suggesting sufficient reduction of portal pressure by the taken measures.

Despite histologically confirmed F4 fibrosis, splenomegaly and even the presence of first-degree varices in the most recent EGD, ARFI did not detect elevated spleen stiffness in patient 7. In five patients, no valid spleen measurements were obtainable (Fig. 1).

Taken together, in contrast to ARFI of the liver, splenic ARFI was not convincingly associated with the presence of esophageal varices in patients with ciliopathies.

### Association of clinical parameters with ARFI results

Next, we analyzed clinical and laboratory parameters and ARFI results by comparing patients with overall fibrosis on ARFI against those with no fibrosis. Regarding laboratory results, ALT, AST, GGT and APRI (aspartate transaminase to platelet ratio index) were significantly higher in the overall fibrosis subgroup whereas platelet counts were significantly lower (Table 1; *p* < 0.001 for APRI and GGT, *p* = 0.001 for AST, *p* = 0.015 for ALT and *p* = 0.007 for platelets). Hepatomegaly and splenomegaly were significantly more common in the overall fibrosis subgroup (Tables 1 and 11% vs. 28% for hepatomegaly, 19% vs. 38% for splenomegaly). Clinically overt portal hypertension only occurred in the F4 fibrosis subgroup (6 patients). With only one child exceeding ARFI cut-offs for F4 fibrosis, children were underrepresented in the overall fibrosis subgroup and therefore the mean age in the subgroup without fibrosis was significantly lower (Table 1). Sex, AP levels and renal replacement therapy were distributed equally between subgroups (Table 1).

### Predictive value of clinical parameters for the presence of overall liver fibrosis

**Fig. 2:**
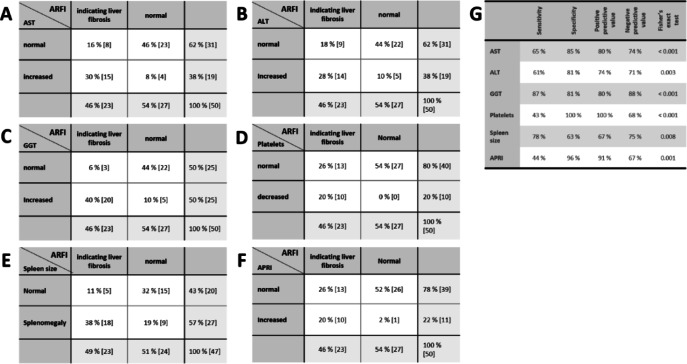
Comparison of different indicators of liver involvement in ciliopathies. Contingency tables showing the relation of liver ARFI results with AST, ALT, GGT, APRI, platelets and splenomegaly (for reference values refer to methods). ARFI values exceeding 1.34 m/s in liver were assessed as indicators for liver fibrosis (equaling F2-F4 fibrosis in adults and F1-F4 fibrosis in children). Panel G: comparison of diagnostic accuracy for different indicators of liver involvement defined by liver ARFI.

Furthermore, we calculated the potential predictive value of individual clinical and laboratory parameters for the presence of significant overall liver fibrosis on ARFI making use of contingency tables and ROC curves (Fig. 2A-F).

Fisher’s exact tests showed a significant association between liver fibrosis and increased AST-, ALT-, GGT-values, APRI, spleen size and decrease of platelets (Fig. 2G). Sensitivity for AST and ALT was only moderate with 65% and 61% whereas specificity was 85% and 81%, respectively. Interestingly, GGT performed better with a sensitivity of 87% and a specificity of 81% (Fig. 2G). However, the positive predictive value of GGT for the detection of overall liver fibrosis was 80%, thus misidentifying a remarkable proportion of non-fibrotic patients when only taking elevated GGT as an indicator (Fig. 2C). In contrast, GGT turned out to be the best parameter to exclude liver involvement with a negative predictive value (NPV) of 88% for all fibrotic patients and even 100% for those with F4 fibrosis. Negative predictive values for ALT, AST, platelet count and spleen size were ≤ 75% and thus not qualifying for excluding underlying liver fibrosis.

Thrombocytopenia with platelet counts < 150.000/µl and APRI only performed with a sensitivity of 43% and 44%, respectively. This is not surprising considering that thrombocytopenia rather reflects patients with portal hypertension than just fibrosis. However, specificity for the detection of ARFI-based fibrosis was 100% and 96%, respectively. For splenomegaly however, only moderate sensitivity (78%) and specificity levels (63%) were calculated (Fig. 2G).

**Fig. 3:**
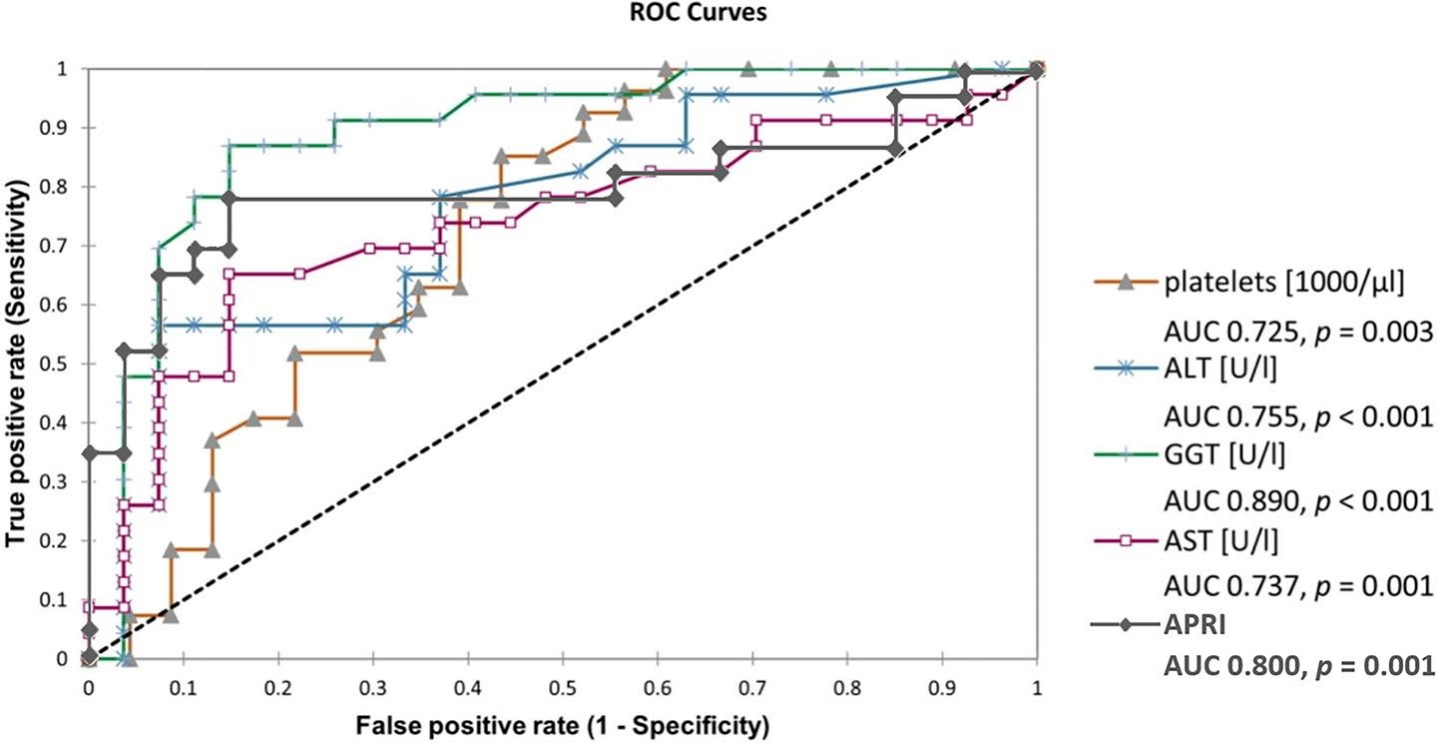
Receiver operating characteristics (ROC) curves for prediction of ARFI-based liver fibrosis by liver enzymes (AST, ALT, GGT), APRI and platelet levels. Area under curves (AUC) and p-values were calculated for each parameter. ARFI values exceeding 1.34 m/s in liver were assessed as indicators for liver fibrosis (equaling F2-F4 fibrosis in adults and F1-F4 fibrosis in children).

Accordingly, GGT showed the highest AUROC value of 0.890, followed by APRI (0.800), ALT (0.755), AST (0.737) and platelets (0.725), (Fig. 3).

In summary, neither elevated liver enzymes nor thrombocytopenia and splenomegaly showed sufficient predictive values to serve as a reliable screening tool for the detection of ARFI-based liver fibrosis in ciliopathy patients.

### Association of genetic variants with liver fibrosis detected by ARFI

**Fig. 4:**
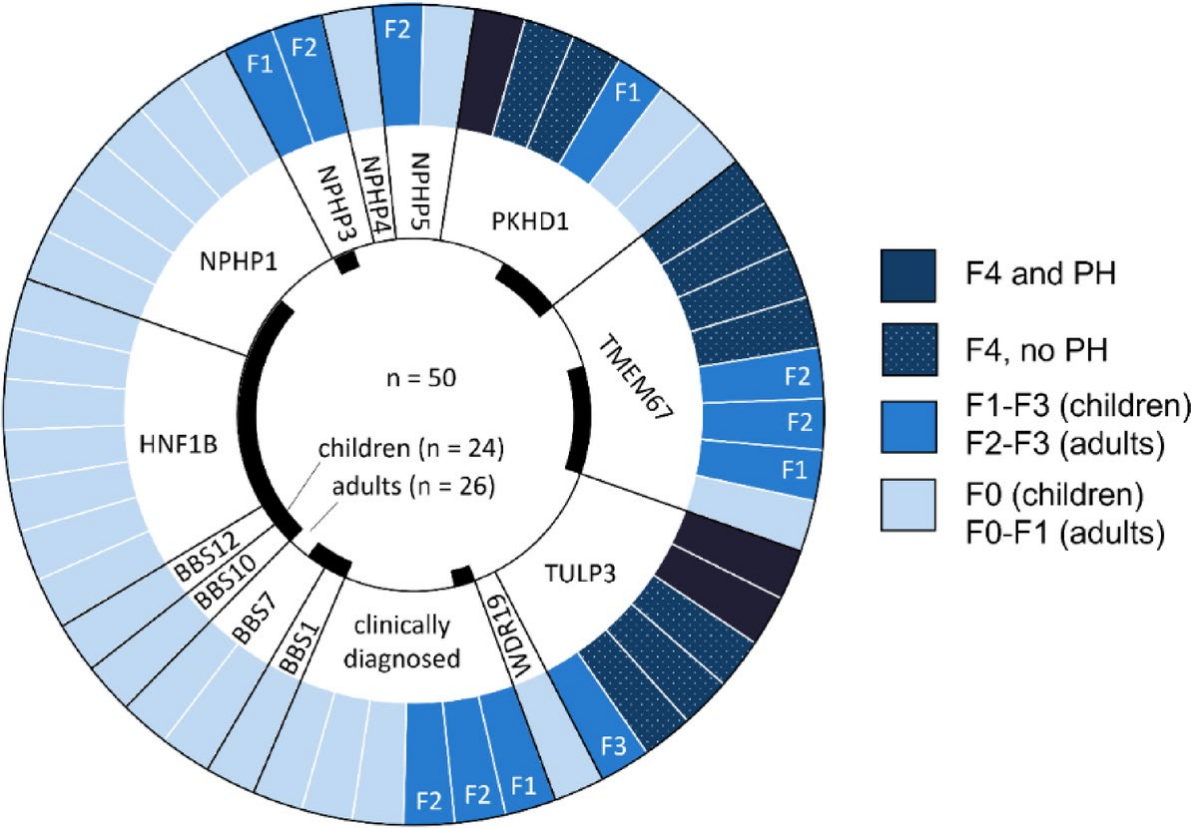
Specific genes are associated with ARFI-based liver fibrosis in ciliopathies. Each bar represents an individual patient grouped by affected gene and coded by ARFI measurements of liver and spleen. Portal hypertension (PH) was defined as pathologic splenic ARFI.

Liver fibrosis detected by ARFI measurement was associated with variants in *PKHD1*, *TMEM67* and *TULP3*, whereas variants in *BBS1*, *BBS7*, *BBS10*, *BBS12*, *HNF1B*, *NPHP1*, *NPHP4* and *WDR19* showed normal liver stiffness (Fig. 4). However, three children (aged 3 years, 7 years and 21 months) with variants in *PKHD1* and *TMEM67* did not show increased liver stiffness. Two patients with variants in *NPHP3* had mild liver fibrosis. *NPHP5* was associated with liver fibrosis in one patient, while one showed ARFI values below cut-off values for F2 fibrosis.

## Discussion

Large phenotypic variability is a central aspect of ciliopathies, making individualized counseling with regard to expectable organ involvement difficult^19,27^. At the same time, early diagnosis of clinically unperceived liver involvement in this disease group can be of vital importance as delayed management with the development of portal hypertension can have devastating effects. However, invasive fibrosis screening by liver biopsy is often not feasible, particularly in pediatric patients. This is also evident in our own cohort in which only 10 out of 50 patients (only adults) had undergone liver biopsy for the histological evaluation of liver fibrosis. On the other hand - despite the limited numbers - liver biopsy results of these 10 patients provided an unique opportunity to compare non-invasive ARFI results with histological observations – a methodological feature that was not available in other studies that examined different elastography systems in the context of ARPKD^18,28–30^. Hartung et al. compared ARPKD patients with healthy controls by ARFI and magnetic resonance elastography (MRE) and suggested discriminating cut-off values for each method^18,28^. In a small study by Kummer et al. FibroScan^®^ was evaluated in ARPKD patients (*n*= 7) versus ADPKD patients and healthy controls. ARPKD patients showed significantly increased liver stiffness compared to controls and ADPKD patients and proved superior to B-mode evaluation of liver fibrosis^29^. Interestingly, Wicher et al. described normal FibroScan^®^results in about a quarter of pediatric ARPKD patients (5/21). However, liver stiffness was still significantly elevated in ARPKD patients compared to controls^30^. This study used elastography cut-off values from another study in which FibroScan^®^was validated by liver biopsies of patients with pediatric chronic liver disease^31^.

Due to comparison of histological fibrosis staging with ARFI results in a subgroup of our patients, we were able to show that ARFI measurements matched histology results perfectly. We have to admit that it is problematic to consider ARFI as a surrogate “ gold standard” for histology-based liver fibrosis when evaluating diagnostic accuracy of various serum markers. Thus, our study has a rather exploratory character. However, it is impossible to obtain liver biopsies of non-fibrotic patients for ethical reasons. This also prevented the development of own ARFI cut-off values for the detection of fibrosis. ARFI elastography in ciliopathy patients seems to identify more “fibrotic” patients than other surrogate markers. It cannot be proven yet that ARFI results better reflect fibrosis, but analogous to better performance of ARFI versus serum markers in hepatitis C patients validated by biopsy, this seems likely^32^.

Other surrogate parameters have traditionally been used to define liver involvement in ciliopathies, such as hepatomegaly, splenomegaly, low platelet count, ultrasound liver echogenicity or elevated liver enzymes^27,33–35^. However, indicators for portal hypertension such as splenomegaly or a low platelet count are of limited use for fibrosis screening as they only occur late in the disease course when portal hypertension is already established. Consistent with this context, our study demonstrated a low platelet count to be very specific for the detection of liver fibrosis, however with a poor sensitivity at the same time.

Screening for liver involvement by the presence of “splenomegaly” can neither be recommended as both, specificity and sensitivity, were low, too. Also, evaluation of liver echogenicity is of limited value, as this parameter is highly subjective on one hand and did not show sufficient sensitivity on the other^36^. According to our data, elevation of transaminases was relatively specific for detection of fibrosis. However, sensitivity turned out to be low with 61 and 65%, respectively. Elevated GGT levels however, performed with a much better sensitivity compared to transaminases with equivalent specificity values. Even more, when restricting the view to ARFI-based F4 fibrosis only, elevated GGT turned out to be the best parameter for excluding liver fibrosis in ciliopathy patients with a negative predictive value of 100%. This observation might be explained by the matter of fact that ciliopathy associated liver disease in some entities is primarily characterized by bile duct alterations (ductal plate malformation) causing dominant elevation of cholestatic enzymes. Interestingly, in a large cohort of patients with Joubert syndrome a predominant elevation of GGT compared to other liver enzymes was also observed^37^.

Whilst ARPKD patients mostly have normal liver enzyme levels^33,34^, the majority of patients displaying a Joubert syndrome with hepatic involvement is characterized by elevated liver enzymes^37^. However, particularly regarding detailed phenotypic information of hepatic involvement in ciliopathies, there is a great and so far unmet need for longitudinal data. Among others, this issue is currently covered by the NEOCYST research initiative (www.neocyst.de).

Screening for esophageal varices by spleen elastography could theoretically help to avoid invasive screening by EGD. Yet, our data suggest that splenic ARFI was too unreliable for variceal screening. However, it must be mentioned that the number of patients with clinically significant portal hypertension was very low in this cohort (6 out of 50 patients). Furthermore, in some of those patients portal hypertension had been treated by TIPS or Warren Shunt before ARFI measurement with an assumed impact on splenic stiffness^38^. Another limitation of this study is the matter of fact that splenic ARFI cut-off values have never been validated in children. Establishing our own cut-off values for the detection of portal hypertension by splenic ARFI was not feasible due to low patient numbers. There is only one small study on children with biliary atresia in which splenic ARFI was suggested to have a predictive value in the screening of esophageal varices^39^.

The concept of non-invasive screening for esophageal varices by elastography still seems to be promising^40,41^ and has to be studied in larger cohorts. Spleen stiffness measurement by FibroScan^®^has been studied much more often in this context^42^especially in pediatric cohorts^43,44^. These studies have suggested Fibroscan to be helpful in non-invasvie screening for esophageal varices.

Our study suggests an association of *PKHD1*genotype with liver fibrosis, which is an obligate finding in ARPKD^45^. *TMEM67* and *TULP3*-associated ciliopathies were frequently associated with ARFI-based liver fibrosis which is in line with previous reports^6,46,47^. Knowledge of genotype in ciliopathies is very helpful as it may lead to a more intensive fibrosis screening of patients at risk. Interestingly, not all patients with *PKHD1* and *TMEM67* variants showed elastographic signs of liver fibrosis. This is in line with a study by Wicher et al. evaluating FibroScan^®^in ARPKD patients which demonstrated normal liver elastography results in about a quarter of patients^30^. As liver fibrosis is considered an obligatory feature of ARPKD, ARFI may not detect all fibrotic patients at all ages. It is not unlikely that liver fibrosis might reach the threshold of detection only later in life in these patients, which can be evaluated by longitudinal elastography measurements.

In HNF1B deficiency^48^and Bardet-Biedl Syndrome^49^liver involvement is not obligatory and has been described to occur only occasionally. In our study we did not show advanced fibrosis stages for these genotypes. Importantly, mechanism of liver injury in BBS may sometimes differ from other mentioned ciliopathies as these patients may also suffer from metabolic syndrome with liver steatosis and potentially steatohepatitis^49^.

In Nephronophthisis 3 (*NPHP3*), liver involvement seems to occur to a relevant extent^50,51^, however in our study only minor fibrosis (F1-F2) was identified in two patients. This result can be explained by low patient numbers and the fact that liver involvement is not obligatory in Nephronophthisis 3.

Limitations of our study are the heterogenous cut-off values for liver fibrosis in children and adults. Another limitation is the missing systematical exclusion of other causes and cofactors for liver fibrosis. Only chronic viral hepatitis was excluded in all patients of the cohort. Observed differences in no fibrosis and overall fibrosis subgroup (Table 1) may in part be explained by selection bias. Many adult patients were recruited from the hepatology section, which made hepatic involvement almost predestined. Recruitment of pediatric patients took place in the pediatric nephrology section, making hepatic involvement much less likely. As all ARFI-based F4 patients were adults except one, conclusions of ARFI screening of liver fibrosis in pediatric patients are limited. Validity of ARFI screening for lower fibrosis stages is also reduced given the fact that only one liver biopsy was available for confirmation of elastography in this subgroup. A further limitation for the comparison of histology-based versus elastography-based fibrosis staging is a long time interval (mean 88.1 months) between liver biopsy and ARFI measurement in a subset of 6 patients.

In conclusion, liver fibrosis screening by ARFI in patients with ciliopathies is a valuable complement to other laboratory and ultrasound surrogate parameters. Spleen stiffness screening by ARFI is not sufficient to reliably exclude esophageal varices. Knowledge of genotype in ciliopathies is helpful in identifying patients at risk for liver fibrosis. However, liver elastography should not replace genetic testing.

To our knowledge, this study is the largest of its kind, assessing patients with mostly genetically determined renal ciliopathies by elastography for potential liver involvement. Early detection of liver fibrosis is of vital importance for this disease group to avoid life-threatening complications like variceal bleeding. This is of particular importance against the background of upcoming causative treatment options for this group of diseases, which however all address kidney survival^52,53^. In this setting, we were able to show that ARFI provides a non-invasive ultrasound-based tool that can help clinicians to not only detect but also monitor liver fibrosis in ciliopathy patients.

## Data Availability

The data that support the findings of this study are available on request from the corresponding author. The data are not publicly available due to ethical / privacy restrictions.

## Abbreviations

ARPKD: Autosomal recessive polycystic kidney disease
MGS: Meckel Gruber syndrome
BBS: Bardet Biedl syndrome
PH: Portal hypertension
ARFI: Acoustic radiation force impulse
NEOCYST: Network for early onset cystic kidney disease
NPH-RC: Nephronophthisis-related phenotype
JS: Joubert syndrome
CKD: Chronic kidney disease
PCR: Polymerase chain reaction
ACMG: American college of medical genetics and genomics
ALT: Alanine aminotransferase
AST: Aspartate transaminase
EGD: Esophagogastroduodenoscopy
GGT: Gamma-glutamyltransferase
EFSUMB: European Federation of Societies for Ultrasound in Medicine and Biology
IQR: Interquartile range
ROC: Receiver operating curve
AUROC: Area under receiver operating curve
TIPS: Transjugular intrahepatic portosystemic shunt
APRI: AST to platelet ratio index
NPV: Negative predictive value
MRE: Magnetic resonance elastography
